# Prodromal Huntington Disease as a Model for Functional Compensation of Early Neurodegeneration

**DOI:** 10.1371/journal.pone.0114569

**Published:** 2014-12-26

**Authors:** Kathrin Malejko, Patrick Weydt, Sigurd D. Süßmuth, Georg Grön, Bernhard G. Landwehrmeyer, Birgit Abler

**Affiliations:** 1 Department of Psychiatry, Ulm University, Leimgrubenweg 12–14, 89522, Ulm, Germany; 2 Department of Neurology, Ulm University, Oberer Eselsberg 45, 89081, Ulm, Germany; Duke-NUS Graduate Medical School, Singapore

## Abstract

Functional compensation demonstrated as mechanism to offset neuronal loss in early Alzheimer disease may also occur in other adult-onset neurodegenerative diseases, particularly Huntington disease (HD) with its genetic determination and gradual changes in structural integrity. In HD, neurodegeneration typically initiates in the dorsal striatum, successively affecting ventral striatal areas. Investigating carriers of the HD mutation with evident dorsal, but only minimal or no ventral striatal atrophy, we expected to find evidence for compensation of ventral striatal functioning. We investigated 14 pre- or early symptomatic carriers of the mutation leading to HD and 18 matched healthy controls. Participants underwent structural T1 magnetic resonance imaging (MRI) and functional MRI during a reward task that probes ventral striatal functioning. Motor functioning and attention were assessed with reaction time (RT) tasks. Structural images confirmed a specific decrease of dorsal striatal but only marginal ventral striatal volume in HD relative to control subjects, paralleling prolonged RT in the motor response tasks. While behavioral performance in the reward task during fMRI scanning was unimpaired, reward-related fMRI signaling in the HD group was differentially enhanced in the bilateral ventral striatum and in bilateral orbitofrontal cortex/anterior insula, as another region sensitive to reward processing. We provide evidence for the concept of functional compensation in premanifest HD which may suggest a defense mechanism in neurodegeneration. Given the so far inevitable course of HD with its genetically determined endpoint, this disease may provide another model to study the different aspects of the concept of functional compensation.

## Introduction

Adult-onset neurodegenerative diseases are supposedly preceded by an extended prodromal phase [1], which is of great interest as it provides an opportunity for insights into disease pathogenesis. Huntington disease (HD), characterized by an adult-onset movement disorder (chorea), behavioural abnormalities and cognitive decline, is particularly well suited for studies of the presymptomatic phase as genetic testing allows for the unequivocal identification of individuals who will become symptomatic [2]. Previous studies of prodromal HD suggested that structural imaging, particularly of striatal volume, is among the most useful read-outs for tracking the prodromal stage [2], [3].

Functional imaging in people at genetic risk for Alzheimer disease revealed increased circuit activation in risk carriers versus controls without differences in actual task performance or marked atrophy [4]. This suggests that in the prodromal phase affected brain regions compensate impending functional deficits by mobilizing a certain activity reserve and that this compensation can be investigated as an even earlier marker of disease progression than actual atrophy.

For HD, functional compensation can be hypothesized for brain regions with intense dopaminergic neurotransmission which show the earliest neuropathological signs of degeneration, specifically in the dorsal, and subsequently in the ventral striatum [5] Accordingly, initially impaired dorsal striatal functioning (punishment avoidance) was accompanied by still intact ventral striatal functioning, i.e. reward learning [6]. We hypothesized that beginning but still subliminal degeneration of ventral striatal neurons in HD should be reflected by correlates of functional compensation in blood oxygenation level dependent (BOLD) signalling as measured with fMRI along with preserved functioning in prodromal gene carriers relative to controls. Ventral striatal functioning was investigated by using a well-established monetary incentive task during fMRI [7], [8]. We predicted signs of ventral striatal functional compensation in prodromal mutation carriers showing no or still minimal ventral striatal but already dorsal striatal structural changes as evidence of beginning neurodegeneration. Notably, to fulfil an important criterion when defining functional compensation we also expected unaffected behavioural performance in the reward task in the HD group as compared to healthy controls.

## Materials and Methods

### Participants

32 subjects completed the experiment and were included in the study. Of these, 14 subjects were carriers of the mutation leading to HD. Sample characteristics are summarized in Table 1. According to their scores in the Unified Huntington Disease Rating Scale (UHDRS) and as defined in Tabrizi et al. [9], eleven were presymptomatic with motor scores lower than 5 [10]. Three subjects had finally to be diagnosed as already early symptomatic, however with disease duration of less than 6 months. The median UHDRS total motor score of the entire sample was 1. All subjects in the HD group were outpatients in the Department of Neurology at Ulm University. This group was matched to a group of 18 healthy controls (CON) for age, gender and education. Data from two more subjects initially enrolled could not be included due to anxiety in the scanner in one case. The other subject did not respond within the expected time interval in the fMRI task (beyond two standard deviations from the mean of the group). Symptoms of HD were further assessed in a detailed interview and a standardized general and neurological examination by an experienced neurologist (co-author PW). Substance related problems and clinical depression were excluded in a psychiatric interview on the basis of the Structured Clinical Interview for DSM-IV (SCID) [11] in mutation carriers and controls. To further assess subclinical symptoms of depression in all participants, the German version of the Center for Epidemiologic Studies Depression Scale, CES-D [12], the ADS (Allgemeine Depressions-Skala) [13], were administered. The DemTect [14] test was used to screen for symptoms of cognitive impairment in all subjects. None of the subjects in either group reached the cut-offs for depression or dementia. The German version of the Sensation Seeking Scale, version V, SSS-V [15], [16], [17] was used to evaluate subclinical group differences in impulsive behaviors, namely thrill and adventure seeking, experience seeking, disinhibiton and boredom susceptibility (Table 1). Participants were asked to refrain from any psychotropic medication including analgesics and sleeping pills for at least 5 days before scanning. Three subjects of the HD group had paused regular CNS medication for participation in the study. One took 15 mg of mirtazapine, one 25 mg of agomelatine, and one 25 mg of agomelatine in addition to 225 mg of venlafaxine. None of these subjects reported adverse symptoms from discontinuation, particularly no agitation or sleeplessness. The study was approved by the local ethics committee of Ulm University. Written informed consent was obtained after complete description of the study and prior to inclusion.

**Table 1:** Sample characteristics.

	HD group	CON group	HD vs. CON	
	n =	n =		
Sex (male/female)	5/9	7/11		
Education (qualified/not qualified for higher education)	9/5	11/7		
Handedness (right/left)	12/2	18/0		
	**mean (SD)**	**mean (SD)**	**t(30)**	***p***
Age (years)	37.1 (8.0)	36.1 (7.7)	0.37	*0.71*
CAG length	42.1 (2.2)			
Estimated time to disease onset (years)	22.1 (12.1)			
SSS-V: Thrill and adventure seeking	6.9 (3.0)	6.8 (2.0)	−0.10	*0.92*
SSS-V: Experience seeking	5.3 (1.4)	6.1 (1.6)	1.20	*0.25*
SSS-V: Disinhibition	3.6 (2.6)	3.6 (2.3)	−0.10	*0.92*
SSS-V: Boredom susceptibility	3.1 (1.6)	3.2 (1.8)	0.13	*0.90*
SSS-V total	19.3 (5.8)	19.7 (5.0)	0.20	*0.84*

SD: standard deviation, HD: Huntington disease mutation carriers, CON: control subjects, t: t-value, p: p-value; estimated time to disease onset: calculated in 11 presymptomatic subjects.

### Task during fMRI scanning

We used a well established monetary incentive task described elsewhere in detail [7], [8] with a variation of probabilities (0%, 25%, 50%, 75%, 100%) to win a fixed amount of money (1 Euro) resulting in parameterized levels of reward value defined as the product of reward probability times magnitude. Within two consecutive sessions, separated by a short break, subjects performed 12 trials per each of the five probabilities, totaling 120 trials. Each trial started with a cue (a colored symbol) that indicated the probability to win the money later on. After an expectation period, subjects had to correctly react with a button press to one of two different symbols. In reacting correctly they preserved the previously announced chance to win one Euro. Feedback (outcome) followed the targets disappearance and notified subjects the amount of money (1€ or 0€) won in the trial. Reaction times and errors were registered. Corresponding to the announced reward probabilities, subjects were not rewarded despite pressing the correct button in a number of trials, i.e. a reward announced at a probability of 75% was actually distributed in 75% (receipt of reward) and held back in 25% (omission of reward) of the correct trials. Incorrect button presses resulted in a feedback of zero Euros at any probability. Receipt and omission trials as well as the five trial types (0–100% chance to win) appeared in random order.

### fMRI acquisition

A 3.0 Tesla MR scanner (Siemens MAGNETOM Allegra, Erlangen, Germany) was used to perform T1 anatomical imaging (1×1×1 mm voxels) and fMRI similar to previous experiments [7], [8]. Functional time series were recorded using a T2*-sensitive gradient echo sequence measuring changes in BOLD-contrast. 23 transversal slices were acquired with an image size of 64×64 pixels. Slice thickness was 3 mm with 0.75 mm gap resulting in voxel sizes of 3×3×3.75 mm. Images were centered on basal structures of the brain including subcortical regions of interest (basal ganglia and prefrontal regions). 401 volumes were obtained during each session at a repetition time (TR) of 1500 ms and echo time (TE) of 35 ms.

### fMRI analysis

Image processing and statistical analysis were carried out using Statistical Parametric Mapping (SPM 8, Wellcome Trust Centre for Neuroimaging, London, UK).

Structural T1 data were analyzed following the standard procedure as described in the VBM tutorial (http://www.fil.ion.ucl.ac.uk/~john/misc/VBMclass10.pdf) for SPM 8 using the diffeomorphic anatomical registration through exponentiated lie algebra (DARTEL) toolbox implemented in the software. T1 images of each subject in native space were segmented into gray matter (GM), white matter (WM) and cerebrospinal fluid (CSF). GM and WM images were iteratively processed by nonlinear registration of the DARTEL toolbox resulting in a final template. Corresponding deformation fields were used to match each gray matter image to this template. Affine transformations were used to register the final DARTEL template to the Montreal Neurological Institute (MNI) space and to warp the individual images into that standard space. Spatial smoothing was applied with a Gaussian kernel of 8 mm full-width at half-maximum (FWHM). The subjects' GM images were entered in two-sample t-tests to assess group differences thresholded at p<0.001 (uncorrected).

Preprocessing of individual functional time series included realignment to correct for motion artifacts, slice timing, spatial normalization via DARTEL into standard MNI space, and smoothing with an 8 mm FWHM Gaussian kernel. Intrinsic autocorrelations were accounted for by first-order autocorrelation modeling and low frequency drifts were removed via high pass filtering.

For individual first level analysis, we defined regressors to analyze each of the five types of expectation phases sorted by reward probabilities, as in our previous studies [7], [8]. Regressors modeled reward expectation including presentation of the cue, the button press and the eight different types of outcome depending on the preceding reward expectation (0–100%) and actual outcome (receipt or omission of reward). According to their actual durations, trials were modeled as timely extended events and convolved with the hemodynamic response function. The six realignment parameters modeling residual motion were also added to the individual models.

The contrast images of parameter estimates for each level of expectation were used to calculate whole brain analyses in both mutation carriers and healthy control subjects and to extract each subject's fMRI signal in the predefined regions of interest (ROIs). Group analyses were calculated on the signal extracted from the ROIs.

### ROI definition

For ROI definition we used peak voxel coordinates of those activations from one of our previous studies with the same task [7] that had been shown to be sensitive to dopaminergic modulation upon expectation of rewards. MNI coordinates of the previously found ventral striatal activation (x/y/z = −18/20/−8) and the anterior insula/lateral orbitofrontal cortex (AI/OFC) activation (x/y/z = 44/26/−12) were used as a starting point. We then defined a 5 mm sphere around this maximum for the left ventral striatum ROI (number of voxels = 81 for functional and 175 for structural images) and an 8 mm sphere for the right AI/OFC ROI (number of voxels = 257 for functional and 583 for structural images). The resulting ROIs were mirrored to the opposite hemisphere (5 mm sphere around x/y/z = 18/20/−8 and 8 mm sphere around x/y/z = −44/26/−12; same number of voxels).

From each subject, the estimated mean fMRI signal averaged across voxels of each ROI was extracted and served as individual parameter estimates of modeled effects for each condition of reward expectation. As in our previous experiments, parameter estimates for each condition (0%, 25%, 50%, 75%, 100%) were weighted with a linear contrast and then summed up to model the slope of increasing brain activation related to increasing reward expectation with increasing probabilities. As previous investigations with the same task [7], [8] did not show any laterality effects, mean parameter estimates from the left and right ROIs were pooled by calculating averaged values.

### Psychological testing

After scanning, subjects performed three reaction time (RT) tasks from the Testbattery for Attentional Performance (TAP, Zimmermann and Fimm, Psychologische Testsysteme, Herzogenrath, Germany).

RT1: Simple reaction to a visual stimulus. Subjects were instructed to press a button with the index finger of the dominant hand as fast as possible upon appearance of a white cross on a black computer screen.

RT2: Reaction to a visual stimulus after a warning tone. As in RT1, subjects were instructed to press a button as fast as possible upon appearance of the white cross, but a warning tone was sent out before the cross appeared.

RT3: Divided attention, involving reaction to distinct visual and auditory stimuli at the same time. Subjects had to press a response button whenever in a sequence of alternating tones of a high and a low pitch two consecutive tones were of the same pitch. Simultaneously, white crosses moved within a four by four matrix on a PC monitor. Subjects had to press a response button whenever a square was formed by four of the white crosses.

### Statistics

Behavioural performance in the reward task during fMRI scanning was analysed by means of an analysis of variance for repeated measures on mean reaction times to test for main and interaction effects of the two factors probability (five levels) and group (two levels) in combination with Bonferroni post hoc testing on within-group pair-wise differences.

Group differences in parameter estimates of fMRI signals extracted from the ventral striatal and AI/OFC ROIs were tested on significance by two planned t-tests using STATISTICA 6.0. To infer significant differences the nominal level of p<0.05 was adjusted by a rough false discovery rate (FDR) correction [18] to account for multiple comparisons (FDR-adjusted p = 0.038).

Regarding psychological testing after scanning with three reaction time tasks three two-sample t-tests were applied to test group differences of mean reaction times on significance. Again, the nominal level of p<0.05 was FDR adjusted to infer significant differences while accounting for multiple comparisons (p = 0.033, FDR adjusted).

## Results

### Behavioral performance during fMRI scanning

**Figure 1:**
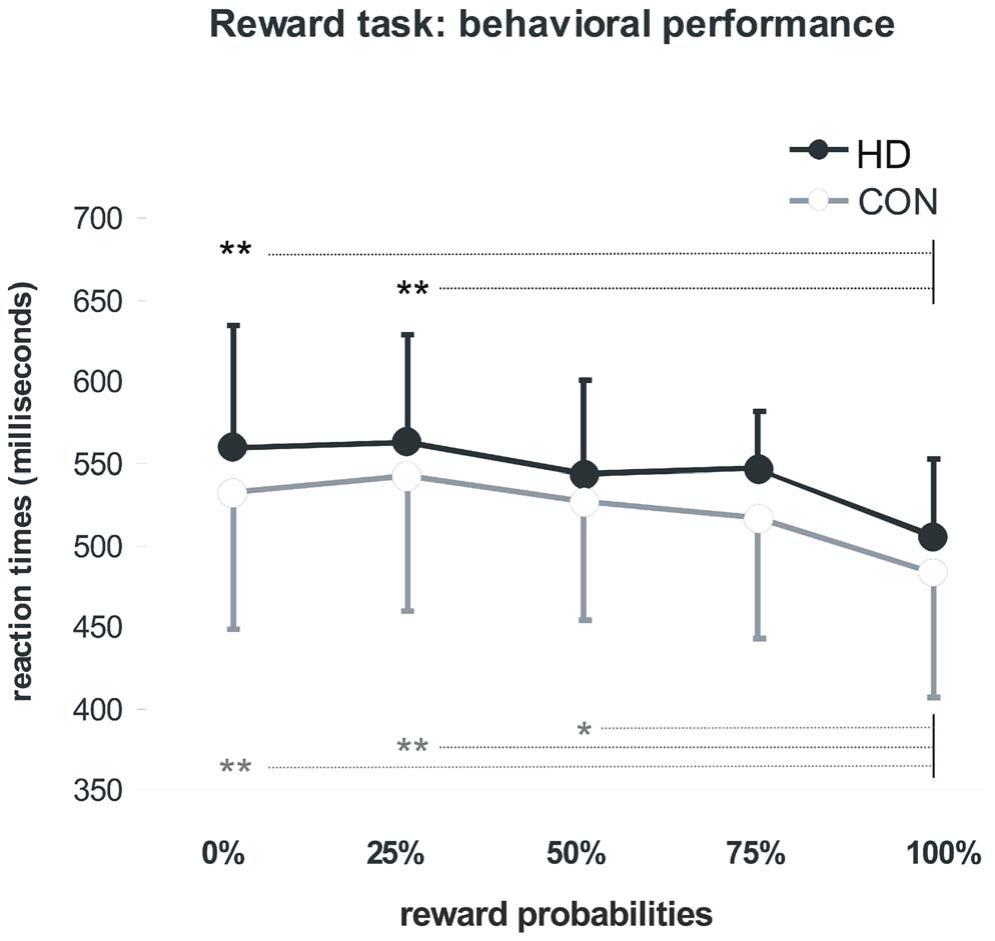
Reaction times: Mean reaction times and their standard deviation in the fMRI reward task. **: p≤0.001 in post hoc testing. *: p<0.005 in post hoc testing. HD: Huntington disease mutation carriers, CON: control subjects.

Subjects pressed the correct button within the required time interval in 97% (HD group) and 99% (CON group) of the trials. An analysis of variance for repeated measures on mean RT revealed a significant main effect for levels of probability (F(4,120) = 15.07, p<0.001), but not for group (F(1,30) = 1.10, p = 0.303) and interaction effects (F(4,120) = 0.20, p = 0.936). The expected acceleration of reaction times upon higher reward probabilities was equally evident in both groups (Fig. 1). Post-hoc tests (Bonferroni) yielded significant acceleration of mean RT when comparing trials with 0% and 25% against trials with 100% probability (0% vs. 100%: p = 0.001; 25% vs. 100%: p<0.001) for the HD group, and a significant acceleration comparing trials with 0%, 25% and 50% against trials with 100% probability (0% vs. 100%: p = 0.001; 25% vs. 100%: p<0.001; 50% vs. 100%: p = 0.004) for the CON group.

### Behavioral performance in neuropsychological tests after scanning

Mean reaction times of the HD group were slower in all three neuropsychological tests. Significant differences were evident for the pure motor test (RT1) with simple reaction upon a visual stimulus. For the other two psychological tests, results showed trends to significance (see Table 2).

**Table 2:** Reaction times of behavioural tasks after scanning.

	HD group		CON group		HD vs. CON		
Reaction Times (ms)	mean	SD	mean	SD	t(30)	d	p
RT1	278	41	240	43	2.53	0.9	0.02
RT2*	260	31	237	39	1.79	0.7	0.08
RT3	704	87	649	73	1.70	0.6	0.06

SD: standard deviation, HD: Huntington disease mutation carriers, CON: control subjects, t: t-value, d: effect size.

Significance was assumed at p≤0.033, FDR adjusted.

*: 1 datapoint missing in 1 subject of the HD group.

### MRI results - structural

**Figure 2:**
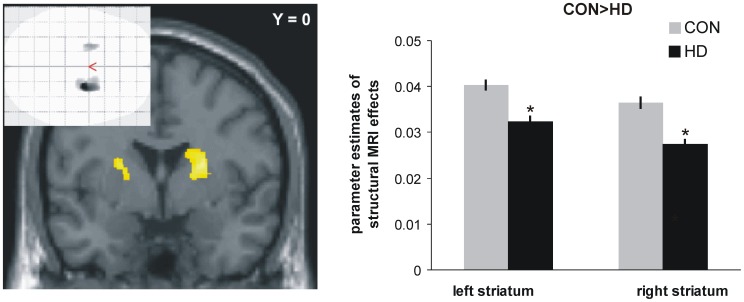
Comparison of structural grey matter images: decreased dorsal striatal volume in mutation carriers as compared to control subjects (*: p<0.001, uncorrected, number of voxels per cluster >20 for illustration purposes). Glass brain figure shows regional specificity of the results. Bar graphs on the right side depict parameter estimates of structural MRI volume differences between HD subjects and healty controls obtained from the left striatal peak voxel (x/y/z = −24/2/15) and the corresponding voxel on the right side (x/y/z = 24/2/15). HD: Huntington disease mutation carriers, CON: control subjects.

Independent two-sample t-tests of whole brain GM images revealed the expected group difference with decreased grey matter volume in dorsal striatal areas in the HD group. No other brain area showed decreases in volume (Fig. 2), and no increases in the HD group relative to controls were observed. However, within the 4 ROIs delineating the predefined parts of the brain's reward system, a trend pointing towards beginning grey matter loss in the HD group was evident with 28 out of 175 voxels in the left ventral striatal ROI and 2 out of the 175 voxels in the right striatal ROI. 83 and 13 voxels out of the 583 voxels in each left and right AI/OFC ROI survived a threshold of p<0.05 for the t-test of CON>HD.

### MRI results - functional

**Figure 3:**
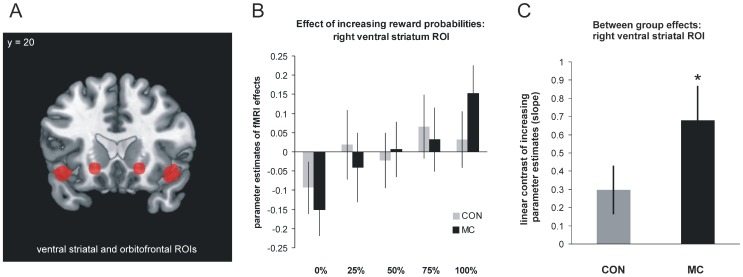
A - Position of 3-dimensional, independent regions of interest (ROIs): ventral striatum and anterior insula/orbitofrontal cortex (AI/OFC). B - Mean corrected parameter estimates of fMRI effect within right ventral striatum ROI: parameter estimates demonstrate increased differential effects of reward expectation in HD compared to the CON group as an expression of functional compensation. C - Linear contrast (slope) of parameter estimates of fMRI effects within right ventral striatum: mean and standard deviation of individual slopes in each group and significant (*: p<0.05) group difference. HD: Huntington disease mutation carriers, CON: control subjects.

The contrast modelling increasing brain activation upon increasing reward expectation confirmed previous findings in both groups separately with a significant (p<0.005) effect in the ventral striatum, AI/OFC and the anterior cingulate cortex. The intended ROI analyses comparing the weighted parameter estimates upon different reward probabilities between both groups revealed significant (p≤0.038, FDR adjusted) differences in the ventral striatal ROI (t(30) = 1.86; p = 0.036; effect size (d) = 0.7) and in the AI/OFC ROIs (t(30) = 2.47; p = 0.010; d = 0.9). In both regions the HD group demonstrated increased differential activation effects relative to healthy controls (Fig. 3). Results remained significant, also after exclusion of the three early symptomatic subjects in the HD group.

Furthermore, a whole brain group comparison showed that apart from the ventral striatal and anterior insula/orbitofrontal regions of interest no differential activation in any other brain region survived corrections for multiple comparisons.

Although groups did not differ on indices of impulsivity as obtained by the SSS-V, correlation coefficients were computed to test on significant associations between individual expressions of this trait and brain activation upon increasing reward expectation within each ROI. Even at lowered thresholds neither the subscales, nor the SSV sum score showed substantial correlations within the group of HD subjects, or across the entire sample of HD subjects and healthy controls (data not shown).

## Discussion

Using structural and functional MRI, we investigated the hypothesis of functional compensation of early neurodegeneration in prodromal Huntington disease. With its genetic determination, this disease may provide a well-suited model to investigate the consequences of early neurodegeneration, and to study aspects of functional compensation, a concept that has been introduced by previous studies on incipient Alzheimer'disease [4], [19]. In this context, beginning degeneration is thought to be compensated by increased activation of the remaining neurons in the same brain region and in a wider sense also by activation of additional parts of cerebral networks resulting in equal behavioural performance between affected and non-affected subjects. In the course of the disease, this compensation may be overridden at some point in time indicated by emerging behavioural deficits and decreased activation due to then markedly decreased number of neurons [20]. As previously demonstrated in other samples of early symptomatic mutation carriers of the Huntingtin gene [9], [21], in the group investigated here, significant structural changes were specifically evident already in dorsal striatal regions while the ventral striatum and also the AI/OFC regions both involved in reward processing showed only very tentative and mild signs of grey matter loss (see also [5]). Positron emission tomography (PET) has shown decreased raclopride binding potentials in the already affected dorsal striatal areas [22], [23] supporting that primarily dopaminergic neurotransmission is involved. Paralleling these structural findings, present behavioural data showed impaired functioning in the HD group when it came to simple reaction time tests like mere responding to a visual stimulus with a button press (RT1). Such simple motor tasks are thought to basically rely on dorsal striatal functioning as confirmed by neuroimaging [24]. Reaction times in the reward task on the other hand were equivalent in mutation carriers and controls. Although the motor response in the reward task most likely requires some recruitment of dorsal striatal neurons, functional imaging has shown that reward related reaction times are most crucially linked to ventral striatal functioning. The acceleration of reaction times observed with increasing reward probability in healthy subjects goes very well along with increased ventral striatal activation [25], [26], [27], [28]. Thus, increased recruitment of ventral striatal neurons in the reward task in the HD group may have compensated for the incipient substance loss and may have contributed to the observed unimpaired behavioural performance. The increased differential effects in fMRI BOLD signalling observed upon reward expectation can be interpreted as a potential correlate for increased ventral striatal neuronal recruitment in the sense of direct functional compensation.

As published previously [7], [8] activation of reward-related brain regions upon reward expectation indicates different reward values with the highest reward values (greatest probabilities to win) being related to the highest activation, the lowest reward values (smallest probabilities) to the lowest activation. Increasing the difference between activation related to low and high reward values as observed in the HD group may help to increase the discriminative capacity upon the preparatory phase and to keep up with faster reaction times for higher value rewards despite of early grey matter loss. The results of a study investigating neural correlates of working memory functioning in presymptomatic mutation carriers [29] can be interpreted in the same way: preserved behavioural functioning in the working memory task was paralleled by increases in activation in brain regions involved in working memory functioning, namely in the left inferior parietal lobule and the right superior frontal gyrus in pre-HD subjects close to onset but with no manifest cortical atrophy [29].

In line with the definition of functional compensation, another published fMRI study of reward processing in carriers of the HD gene did not find any sign of hyperactivation [30]. While in our study group increased activation went along with preserved behavioural functioning, Enzi et al. report significantly slower reaction times in a reward task in mutation carriers, both near and far from onset. This deficient behavioural functioning went along with decreased activation in the group near the onset of the illness while absence of significant activation differences between subjects far from onset and controls was interpreted as a correlate of unchanged neuronal functioning [30]. In the light of our findings, the relatively unchanged activation, however, would not be interpreted as still preserved activation in a preserved number of neurons but rather as hyperactivation in an already decreased number of nerve cells.

The AI/OFC investigated as a second key region of the reward system with reported degeneration relatively early in Huntington disease [9] but commonly only after striatal areas are affected showed the same increased responsiveness upon reward expectation as the ventral striatum. This may support the hypothesis that functional compensation of early structural degeneration is not only relevant for subcortical but also for cortical structures and aligns with other findings like those regarding working memory [29]. However, it may also represent an additional recruitment of cortical regions for compensatory reasons in the same way as Paulsen et al. [31] interpreted activation in medial hemispheric structures (pre-supplementary motor area and cingulate) observed in a time discrimination task as compensation for reduced subcortical activation in mutation carriers.

Although our analyses followed a strict rationale with a priori defined independent regions of interest and corrections for multiple comparisons, statistical strength of between-group comparisons should not be interpreted as confirmatory in a clinical sense. Stronger conclusions certainly await a replication in an independent and larger sample of mutation carriers with greater homogeneity regarding the putative course of the disease. Also, the heterogeneity in the sample with respect to age, education and social status may have contributed to present t-statistics of the between-group comparisons by affecting the variance of neural signalling also in the control group. These limitations should be taken into account when interpreting present data.

Still, the concept of functional compensation appears to emerge as a common compensatory mechanism in different sporadic and inherited neurodegenerative diseases. Analogous to hippocampal hyperactivation in Alzheimer disease [20], hyperactivation of reward-related brain regions could reflect an exitotoxic disease process. As furthermore discussed for fMRI findings of functional compensation in Alzheimer disease [19], decreased basal metabolic rates may explain relative fMRI signal increases. In this context, longitudinal investigations are very needed to evaluate the predictive value of the degree of functional compensation for the characterization of further disease development. This would help to further test overall validity of this concept. Longitudinal within-subjects investigations should show that at a certain point in time, grey matter loss in the so far compensating area crosses a critical threshold with the consequence that functional compensation fails and behavioural performance deteriorates.
